# Global crop introduction drives host jumps, turning native pathogens into emerging diseases

**DOI:** 10.1073/pnas.2536984123

**Published:** 2026-05-08

**Authors:** Uma Crouch, Andrew Paul, Ignazio Carbone, Uwe Braun, Bailey Pelt, Gerald Holmes, Susumu Takamatsu, Dan-Ni Jin, Shu-Yan Liu, Michael Bradshaw

**Affiliations:** ^a^Center for Integrated Fungal Research, Department of Entomology and Plant Pathology, North Carolina State University, Raleigh, NC 27606; ^b^Institute of Biology, Department of Geobotany and Botanical Garden, Herbarium, Martin Luther University, Halle (Saale) 06099, Germany; ^c^Strawberry Center, College of Agriculture, Food & Environmental Sciences, California Polytechnic State University, San Luis Obispo, CA 93407; ^d^Professor Emeritus, Laboratory of Phytopathology, Graduate School of Bioresources, Mie University, Tsu, Mie 514-8507, Japan; ^e^Department of Plant Pathology, College of Plant Protection, Jilin Agricultural University, Changchun, Jilin Province 130118, China

**Keywords:** emerging diseases, plant pathogens, host shifts

## Abstract

Global crop movement has traditionally been viewed as a major driver of emerging plant diseases through the introduction of pathogens into naïve environments. Here we show that the reverse process, introducing crops into regions containing endemic pathogens already adapted to related native hosts, is an equally powerful but underrecognized mechanism of disease emergence. Using multilocus phylogeny, haplotype networks, SplitsTree analysis, and molecular clock dating of both fresh and century-old herbarium specimens, we reconstructed the global history of powdery mildews infecting strawberries and raspberries. We reveal that these fungi comprise ancient, geographically structured, host-specialized lineages rather than a single cosmopolitan species as previously assumed. North American lineages infecting strawberries (*Podosphaera shepherdiae*) and Eurasian lineages infecting strawberries (*P. fragariae*) trace their origins to native hosts, predating modern agriculture by millions of years. Raspberry-infecting lineages showed similar patterns of local endemism and host association. These findings demonstrate that emerging plant diseases can arise not only when pathogens move globally, but also when nonnative crops are introduced into landscapes containing long-established native pathogens. This work highlights the importance of taxonomic resolution and herbarium genomics for identifying the true origins of agricultural diseases and for understanding the evolutionary pathways that give rise to modern epidemics.

Understanding the dynamics of pathogen populations is essential for preventing the emergence and spread of plant diseases. Historically, many devastating epidemics have resulted from the movement of pathogens. A classic example is the oomycete *Phytophthora infestans*, the causal agent of the Irish potato famine, which is hypothesized to have originated in the Andes (1). Similarly, the powdery mildew *Erysiphe vaccinii* spread from North America to new regions causing yield losses to the blueberry industry (2). In both cases, the pathogen itself was introduced into naïve environments, leading to severe disease outbreaks.

Introduction of cultivated hosts to new geographic regions can also result in native pathogens causing new diseases on these introduced plants. As obligate biotrophic fungi, powdery mildews provide an ideal system for understanding this process. Such insights can be enabled by mycological herbaria, which serve as invaluable and underutilized repositories of preserved specimens collected across time. Despite their economic importance, powdery mildews have long been taxonomically challenging. Many species were historically grouped under broad concepts that masked underlying evolutionary relationships. Such oversimplified taxonomy can obscure patterns of host specificity, geographic structure, and pathogen origin. This was particularly evident in *Podosphaera aphanis*, the name historically applied to all powdery mildews infecting strawberries and raspberries and assumed to represent a single, globally distributed species. However, initial analyses revealed that this group comprises multiple host-specific lineages with distinct geographic ranges (3).

Our objectives were to identify and delimit the powdery mildew species infecting these high-value crops worldwide and to reassess older, morphologically distinct taxa to determine whether historical host jumps could explain their diversity. Using multilocus phylogeny and population genetics, we examined the origins, geographic distributions, and divergence histories of these pathogens. By analyzing both fresh and century-old herbarium specimens, we reconstructed their evolutionary histories.

## Results

### Sample Collection.

Fresh and herbarium specimens were collected globally. Clear geographic segregation was observed: *Podosphaera fragariae* occurred only in Europe and Asia; *P. shepherdiae* only in the Americas. Powdery mildew species infecting *Rubus* generally mirrored the native distributions of their hosts.

**Fig. 1.**
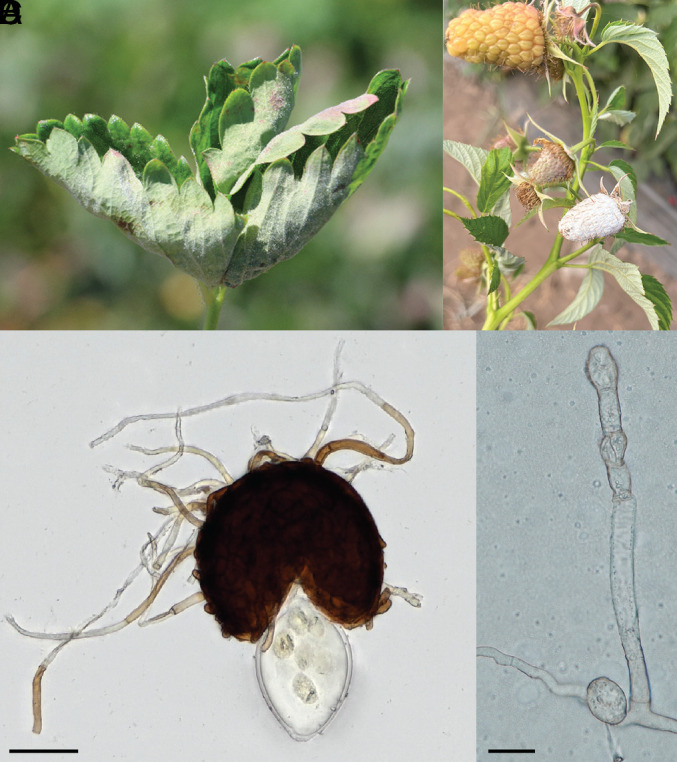
Powdery mildew infecting strawberries and raspberries caused by the fungal species *P. ruborum* and *P. shepherdiae* (*A*) Infected strawberry leaf. (*B*) Infected raspberry fruit (*C*) Sexual, overwintering, stage of powdery mildew caused by *P. shepherdiae* (NCSLG24634). (*D*) Asexual stage of powdery mildew caused by *P. ruborum* (NCSLG25136). (Scale bar, *C* = 50 um, *D* = 25 um.).

Powdery mildew was collected from a range of hosts. Several herbarium specimens dated back to the early 1900s including the type of *P. shepherdiae*. *P. shepherdiae* was first collected and sequenced from Montana in 1913 on *Shepherdia argentea* (two specimens) and on *Glossopetalon spinescens* from Oregon and Idaho in 1926. Additional herbarium specimens identified as *P. shepherdiae* and sequenced in the present study included collections from Argentina (1994, NCSLG24634; 2001, NCSLG25919). These herbarium specimens provided data for confirming the historical occurrence and evolutionary placement of *P. shepherdiae* and allowed comparison with modern collections included in our phylogenetic analyses. Notably, *P. ruborum* f. *californica* was found exclusively on a single proprietary raspberry cultivar grown in southwestern North America (Fig. 1*B*). Although this cultivar is cultivated globally, *P. ruborum* f. *californica* has only been detected on this host in southwestern North America suggesting that this lineage may be indigenous to this region and may have coevolved with one of the many native *Rubus* species endemic to this area of the world.

### Morphology.

Previously, powdery mildew infecting strawberries (*P. shepherdiae* and *P. fragariae*) were grouped under the name *P. aphanis* and thought to be morphologically indistinguishable (3). However, in our reexamination of chasmothecia, we revealed that *P. shepherdiae* has shorter, wider appendages than *P. fragariae* (3, 4) (Fig. 1*C*).

### Phylogeny and population genetics.

**Fig. 2.**
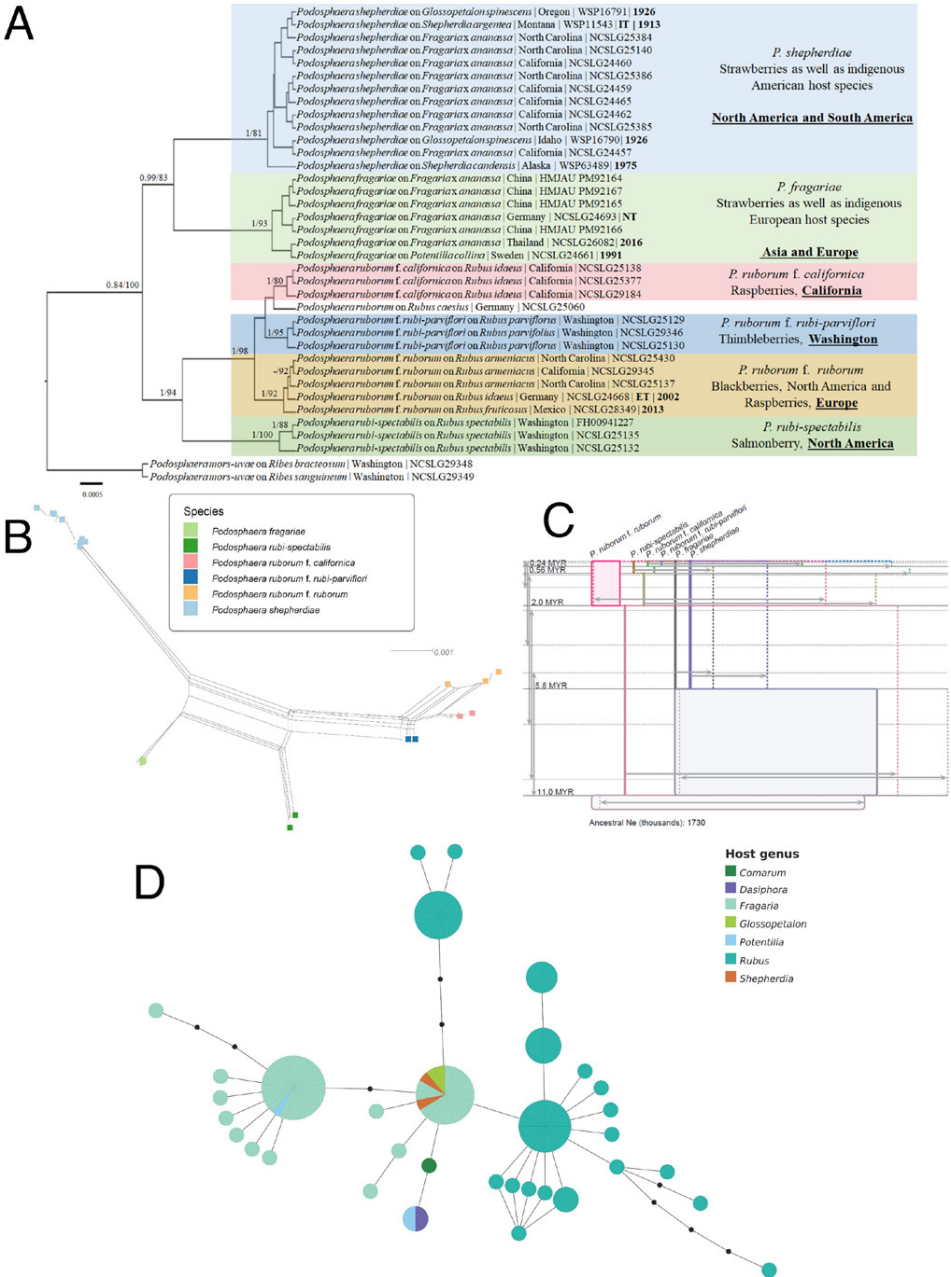
(*A*) Bayesian maximum clade credibility tree inferred from concatenated ITS+28S+*GAPDH*+IGS+*RPB2*+*TUB* sequences of *Podosphaera* taxa. Posterior probabilities are followed by maximum likelihood (ML) bootstrap support values. Type status (IT = Isotype, NT = Neotype, ET = Epitype) is indicated for relevant specimens and the year of collection is in bold for the herbarium specimens. The tree includes specimens with multiple loci successfully sequenced; additional single-locus data, including specimens from South America, are provided in the Dryad repository (5). (*B*) A SplitsTree network resolving six major bipartitions corresponding to the different taxa, with parallel edges indicating recombination primarily within but not among taxa. (*C*) the IMa3 isolation-with-migration population tree with box widths proportional to effective population size (Ne) and 95% CI for Ne and divergence times (MYA); no significant migration was detected, consistent with divergence under geographic isolation. (*D*) the statistical parsimony haplotype network (95% connection limit), which forms a single multilocus network with reticulation. Node pie charts represent haplotype frequencies across host genera revealing clear host-associated patterns in genotype distribution.

Of the 70 sequenced specimens, 35 with multiple loci sequenced were included as references in the multilocus phylogeny (Fig. 2*A*). *P. fragariae*, *P. shepherdiae*, *P. rubi-spectabilis*, and *P. ruborum* each formed well-supported clades, with multiple host-associated subclades within *P. ruborum*. SplitsTree analysis (Fig. 2*B*) resolved six major groups corresponding to the focal powdery mildew taxa, and the statistical parsimony haplotype network (Fig. 2*D*) revealed strong clustering of multilocus haplotypes by host genus. The IMa3 maximum posterior tree (Fig. 2*C*) provides divergence times ranging from ~0.24 to 11 MYR, consistent with lineages that predate agriculture and evolved long before anthropogenic influence. No statistically significant migration was detected, indicating divergence under isolation rather than ongoing gene flow, and effective population size estimates further support the demographic distinctiveness of these lineages. Together, these results reinforce the interpretation that host-associated *Podosphaera* lineages represent independently evolving taxa.

## Discussion

Our results underscore the importance of taxonomy in interpreting pathogen diversity and disease dynamics. By integrating molecular and morphological data, we show that what was once considered a single, cosmopolitan species actually represents multiple, geographically and host-delimited taxa. Recognizing this hidden diversity clarifies evolutionary relationships, and improves our understanding of host specificity.

Our analyses revealed that *P. shepherdiae*, which infects North American strawberries, forms a distinct lineage. Sequences from powdery mildew infecting *Shepherdia argentea* and *Glossopetalon spinescens* collected from the early 1900s clustered with those infecting strawberries from North America, supporting a close evolutionary relationship among pathogens infecting native North American hosts and cultivated *Fragaria*. Similarly, *P. fragariae* was noted on two different European native *Potentilla* species, indicating that this lineage likely originated on Eurasian rosaceous hosts before colonizing cultivated strawberries. However, an alternative scenario cannot be discounted: *P. shepherdiae* may have originally evolved on *Fragaria chiloensis*, a wild strawberry species native to the Americas. We sequenced multiple specimens of powdery mildew from *F. chiloensis* collected in South America, and these isolates grouped within the same *P. shepherdiae* clade. In this scenario, *P. shepherdiae* first infected *F. chiloensis* in South America, then shifted to agriculturally bred strawberries, which were later introduced to North America, where the pathogen subsequently colonized native hosts. It should also be noted that *F. chiloensis* occurs naturally in both North and South America and contributed to the origin of cultivated strawberries. The complex evolutionary history and hybrid origin of cultivated strawberries mean that additional evolutionary scenarios for host shifts or pathogen diversification cannot be excluded. Nonetheless, this would still represent an endemic pathogen infecting a nonnative host. Supporting this timeline, MycoPortal records indicate that powdery mildew on *Shepherdia* or *Glossopetalon* was not collected until 1894, after the first documented cases of strawberry powdery mildew in the United States which was first recorded in 1886 in New York and 1892 in Massachusetts (6). However, the absence of earlier records on these hosts may also reflect incomplete historical sampling rather than a true absence of the pathogen.

Powdery mildew infections on raspberries (*P. ruborum)* displayed a similarly strong relationship between pathogen distribution and host geography (Fig. 2). Molecular clock analyses indicate that these lineages are ancient, with raspberry-infecting powdery mildews diverging approximately 240 thousand years ago and strawberry-infecting species around 5.8 Mya, well before any anthropogenic influence (Fig. 2*C*). Our analysis indicates that any recombination detected is ancient and consistent with the evolution of strict host specificity. However, given the global trade and agricultural movement of strawberries, these pathogens (*P. fragariae* and *P. shepherdiae*) could eventually co-occur in the same geographic locality and infect the same host (*Fragaria*), potentially enabling hybridization similar in principle to that described by Menardo et al. (7). Future studies integrating whole-genome sequencing and population genomic approaches will be important for detecting future recombination events and for precisely tracking pathogen introductions and movements across global strawberry production systems.

This study highlights how inclusion of native host sampling enhances understanding of pathogen origins and disease emergence. Emerging diseases can arise not only from pathogen introductions but also from native pathogens adapting to introduced plants (8). Our findings emphasize the value of herbaria and collections that include nonagricultural plants, which provide essential information about pathogen origin and reveal potential reservoirs of inoculum. Integrating native species into studies of agricultural pathogens offers a more complete view of disease population structure and dynamics. Equally important, this work demonstrates that careful taxonomic resolution and a narrow species concept are vital for uncovering hidden evolutionary relationships and understanding the true diversity of pathogens that shape agricultural systems worldwide.

## Materials and Methods

Seventy powdery mildew specimens infecting strawberries and raspberries were collected globally from both fresh material and historical herbarium specimens spanning the early 1900s to the present. DNA was extracted, PCR was conducted, and multiple loci were sequenced using sanger sequencing. Concatenated phylogenetic analyses, haplotype networks, and SplitsTree approaches were used to evaluate host association, geographic structure, and evidence of recombination. Divergence times, effective population sizes, and migration parameters were estimated using multilocus isolation-with-migration models. Full methodological details, including laboratory protocols, analytical settings, and sequence accession information, are provided in the Supporting Information. All data analyzed in the current study are available through the Dryad Repository (5).

## Supplementary Material

Appendix 01 (PDF)

## Data Availability

Sequencing and phylogeny data have been deposited in GenBank and T-BAS (X4KCKU5F) (9, 10). All study data are included in the article and/or *SI Appendix*.

## References

[1] A. L. Coomber , A pangenome analysis reveals the center of origin and evolutionary history of *Phytophthora infestans* and 1c clade species. PLoS One **20**, e0314509 (2025). 39854309 10.1371/journal.pone.0314509 PMC11760636

[2] M. Bradshaw , An emerging fungal disease is spreading across the globe and affecting the blueberry industry. New Phytol. **246**, 103–112 (2025). 39775676 10.1111/nph.20351

[3] M. Bradshaw , Powdery mildews on *Fragaria* spp. and *Rubus* spp.—unravelling the phylogeny and taxonomy of economically relevant species within the *Podosphaera aphanis* s. lat. complex. Mycoscience **66**, 222–231 (2025). 41971980 10.47371/mycosci.2025.05.001 PMC13062953

[4] M. Bradshaw, A. Paul, U. Braun, Taxonomic supplement to “Global crop introduction drives host jumps, turning native pathogens into emerging diseases”. Schlechtendalia **43**, 15–18 (2026). 10.1073/pnas.2536984123 PMC13167733 42101999

[5] M. J. Bradshaw, Data from: Global crop introduction drives host jumps, turning native pathogens into emerging diseases. Dryad Digital Repository (2026), 10.5061/dryad.ht76hdrxf. PMC13167733 42101999

[6] L. R. Hesler, H. H. Whetzel, Manual of Fruit Diseases—Strawberry Diseases: Powdery Mildew (Macmillan, 1922), pp. 425–426.

[7] F. Menardo , Hybridization of powdery mildew strains gives rise to pathogens on novel agricultural crop species. Nat. Genet. **48**, 201–205 (2016). 26752267 10.1038/ng.3485

[8] T. I. Burgess, M. J. Wingfield, Pathogens on the move: A 100-year global experiment with planted eucalypts. Biosci. **67**, 14–25 (2017).

[9] I. Carbone, J. B. White, T-BAS reference tree datasets [Data set]. T-BAS. 10.52750/634503. Deposited 6 December 2025.

[10] I. Carbone, J. B. White, DeCIFR: an integrated biological data informatics platform [Platform record]. DeCIFR. 10.52750/845633. Accessed 13 December 2025.

