# Supplementary material for: Global crop introduction drives host jumps, turning native pathogens into emerging diseases
Source: Proc Natl Acad Sci U S A. 2026 May 8;123(19):e2536984123. doi: 10.1073/pnas.2536984123 (PMC13167733; doi:10.1073/pnas.2536984123)
Supplement: Supplementary file 1 — Appendix 01 (PDF) [file pnas.2536984123.sapp.pdf]

Supporting Information for Brief Report:

Global Crop Introduction Drives Host Jumps, Turning Native Pathogens into Emerging Diseases

Uma Crouch[1], Andrew Paul[1], Ignazio Carbone[1], Uwe Braun[2], Bailey Pelt[1], Gerald Holmes[3], Susumu Takamatsu[4], Dan-Ni Jin[5], Shu- Yan Liu[5], and Michael Bradshaw[1]
[1]Center for Integrated Fungal Research, Department of Entomology and Plant Pathology, North Carolina State University, 851 Main Campus Drive, Raleigh, North Carolina 27606, USA.
[2]Martin Luther University, Institute of Biology, Department of Geobotany and Botanical Garden, Herbarium, Am Kirchtor 3, 06099 Halle (Saale), Germany
[3]Strawberry Center, College of Agriculture, Food & Environmental Sciences, California Polytechnic State University, San Luis Obispo, CA 93407 USA
[4]Professor Emeritus, Laboratory of Phytopathology, Graduate School of Bioresources, Mie University, Tsu, Mie 514-8507,raduate School of Bioresources, Mie University, 1577 Kurima–Machiya, Tsu, Mie 514-8507, Japan
[5]Department of Plant Pathology, College of Plant Protection, Jilin Agricultural University, No. 2888 Xincheng Street, Changchun 130118, Jilin Province, China
Corresponding Author:
Michael Bradshaw; **Email:** mjbradsh@ncsu.edu,

This PDF file includes:

Supporting text

Supporting Information Text

Materials and Methods

All metadata associated with this manuscript including single locus trees, distribution maps, and specimen data are available via DRYAD (1).

Phylogenetic analyses and species identification

Seventy powdery mildew specimens infecting *Fragaria* and *Rubus* were obtained from fresh collections and historical herbarium material spanning the early 1900s to the present, with sampling from North America, Europe, Asia, and South America. Data included specimens from cultivated strawberries and raspberries as well as from native rosaceous hosts, including *Shepherdia*, *Glossopetalon*, *Potentilla*, *Geranium*, *Dasiphora*, *Ribes*, and *Parietaria*, thereby capturing both agricultural and non-agricultural host associations across the geographic range of these pathogens.

Species were identified via a combination of morphological examination of collected specimens and phylogenetic placement through DNA sequencing. DNA extractions were done using the Chelex method (2,3). Polymerase chain reaction (PCR) was carried out for the ITS+28S, *GAPDH*, *IGS*, *RPB2*, and *TUB* regions with several species-specific and/or region-specific primer pairs listed in Bradshaw et al. (4,5). 193 sequences were deposited in GenBank (PX060806–PX060841 and PX134675–PX134831). All additional *Podosphaera* sequences associated with *Fragaria* and *Rubus* from GenBank (51 sequences) were retrieved. These comparative sequences represent previously published material and together with newly generated data encompass the full diversity of lineages evaluated in this study.

A representative phylogenetic tree was constructed from concatenated ITS+28S+*GAPDH*+IGS+*RPB2*+*TUB* sequences from 37 specimens (Figure 2a) using the methods from Bradshaw et al. (6). *Podosphaera mors-uvae* was selected as the outgroup based on preliminary multilocus analyses, which consistently resolved it as a lineage basal to, but outside of, the highly supported strawberry/raspberry clade within *Podosphaera* (1). The best tree, alignments, and specimen metadata (e.g. host, locality) were uploaded to T-BAS v2.4 (7) to enable real-time phylogeny-based placement of unknown *Podosphaera* strains for any number of the seven loci in the reference tree. The reference tree is available in T-BAS (accession X4KCKU5F) for viewing and sequence placement (8).

Network and Haplotype inference:

Network inference was based on parsimony and neighbor-joining methods implemented in TCS v.1.21 (9) and SplitsTree4 v.4.14.8 (10), respectively, and executed via the DeCIFR platform (11) which hosts both applications. In TCS, sequences were collapsed into haplotypes with gaps treated as missing data. Nodes were colored as a function of host and node size was proportional to haplotype frequency. The Neighbor-net algorithm implemented in SplitsTree was used to further identify splits or bipartitions in the data, where the presence of multiple parallel edges between bipartitions indicates a history of recombination; in the absence of phylogenetic conflict, splits would be separated by a single edge. Recombination among formae was further tested using the pairwise homoplasy index (PHI) implemented in SplitsTree.

Multilocus Isolation with Migration

For each locus, a single recombination-free partition was extracted from the multiple sequence alignment using the four-gamete criterion to identify the largest interval of compatible SNPs, as implemented in the Pop-Gen Pipeline Platform (12), accessed via the DeCIFR platform (11). The best time-ordered rooted topology of taxa was determined by performing topology sampling runs with hyperpriors under an infinite-sites mutation model using IMa3 v. 1.11 (13). Population parameter estimates of migration rates ($2N_em$), splitting times (t), and effective population size (N_e) were performed using the fixed best rooted topology. Markov chain Monte Carlo (MCMC) runs were based on a burn-in of 1,000,000 steps, 400 heated chains using a geometric heating scheme of parameters ha = 0.97 and hb = 0.8, 10,000 sampled genealogies, a mutation rate 1×10^{-9} per base per generation, and a generation time of 1 year. Convergence in parameter estimates was based on two runs each with swapping rates greater than 0.9 and effective sample sizes in excess of 10,000. The best-rooted phylogeny was redrawn to include population

parameter estimates and visualized using the IMfig program (https://github.com/jodyhey/IMa3). All runs were performed using tools within the DeCIFR (11).

SI References

1. Bradshaw MJ (2026) Data from: Global crop introduction drives host jumps, turning native pathogens into emerging diseases. Dryad Digital Repository. doi: 10.5061/dryad.ht76hdrxf.
2. Hirata T, Takamatsu S (1996) Nucleotide sequence diversity of rDNA internal transcribed spacers extracted from conidia and cleistothecia of several powdery mildew fungi. Mycoscience 37:283-288. doi:10.1007/BF02461299
3. Walsh PS, Metzger DA, Higuchi R (1991) Chelex 100 as a medium for simple extraction of DNA for PCR-based typing from forensic material. Biotechniques 10:506-513.
4. Bradshaw M, Braun U, Mitchell J, Crouch U, Thomas J, Pfister DH (2026) Phylogeny and taxonomy of the genera of Erysiphaceae, part 8: *Podosphaera* sect. *Tridactyla*. Mycologia 118: 116-129 doi:10.1080/00275514.2025.2554558
5. Bradshaw MJ, Guan G-X, Nokes L, Braun U, Liu S-Y and Pfister DH (2022) Secondary DNA barcodes (CAM, GAPDH, GS, and RpB2) to characterize species complexes and strengthen the powdery mildew phylogeny. Front. Ecol. Evol. 10:918908. doi: 10.3389/fevo.2022.918908
6. Bradshaw M, Ivors K, Broome JC, Carbone I, Braun U, Yang S, Meng E, Warres B, Cline WO, Moparthi S, Llanos AK, Apaza W, Liu M, Carey J, El Ghazouani M, Carvalho R, Elliot M, Boufford D, Coetzee T, de Wet J, Mitchell JK, Quijada L, Meeboon J, Takamatsu S, Crouch U, LaGreca S, Pfister DH (2025) An emerging fungal disease is spreading across the globe and affecting the blueberry industry. New Phytol. 246:103-112. doi: 10.1111/nph.20351
7. Carbone I, White JB, Miadlikowska J, Arnold AE, Magain N, Miller MA, U'Ren JM, Lutzoni F (2019) T-BAS v.2.1: Tree-based alignment selector toolkit for evolutionary placement of DNA sequences and viewing alignments and specimen metadata on curated and custom trees. Microbiol. Resour. Announc. 8:e00328-19. https://doi.org/10.1128/MRA.00328-19.
8. Carbone I, White JB (2025). T-BAS reference tree datasets [Data set]. T-BAS. doi: 10.52750/634503
9. Clement M, Posada D, Crandall KA (2000) TCS: A computer program to estimate gene genealogies. Mol. Ecol. 9:1657-1659.
10. Huson DH (1998) SplitsTree: Analyzing and visualizing evolutionary data. Bioinformatics 14:68-73.
11. Carbone I, White JB. (2025). DeCIFR: an integrated biological data informatics platform [Platform record]. DeCIFR. doi: 10.52750/845633
12. Webb A, Knoblauch J, Sabankar N, Kallur AS, Hey J, Sethuraman A (2021). The Pop-Gen Pipeline Platform: A software platform for population genomic analyses. Molecular Biology and Evolution 38(8): 3478–3485. https://doi.org/10.1093/molbev/msab113.
13. Hey J, Wakeley J (1997) A coalescent estimator of the population recombination rate. Genetics 145:833-846. doi:10.1093/genetics/145.3.833
